# A Rare Case of HIV-Induced Neutropenia Resulting in Haemophilus influenzae Septic Oligoarthritis: A Case-Based Literature Review

**DOI:** 10.7759/cureus.29081

**Published:** 2022-09-12

**Authors:** Navid Mahabadi, Zaynab Al-Sagri, Ali Ali, Jasleen Kaur

**Affiliations:** 1 Internal Medicine, Wayne State University Detroit Medical Center, Detroit, USA; 2 Rheumatology, Wayne State University Detroit Medical Center, Detroit, USA; 3 Rheumatology, Central Michigan University College of Medicine, Saginaw, USA; 4 Rheumatology, Ascension Medical Group, Saginaw, USA

**Keywords:** hiv, human immunodeficiency virus, oligoarthritis, septic arthritis, infection immunology, clinical immunology, hiv aids

## Abstract

Septic arthritis is a medical emergency that rarely occurs without direct trauma to a joint, compromise or trauma to the synovium, or internal hematogenous seeding from bacteremia. Infection of a single joint space is a cause for concern, and infection of multiple joints is even more rare and concerning. Human immunodeficiency virus (HIV) renders patients particularly susceptible to encapsulated bacteria as it compromises opsonization, humoral immunity, as well as neutrophil function. Neutrophils play an important role in preventing and fighting off infections of the synovium, and it is well documented that compromised neutrophil function can result in this peculiar infection. HIV is popularly acknowledged for its suppression of the lymphoid division of the immune system, particularly CD4 T-cells suppression. However, HIV’s effects on myeloid cells are largely overlooked in medical academia, specifically with respect to neutrophil dysfunction. We will explore a case where compromised neutrophil function results in rare infiltration of *Haemophilus influenzae* resulting in polyarticular septic arthritis.

## Introduction

Polyarticular septic arthritis consists of 15-20% of all cases of septic arthritis and carries a 30% mortality rate compared to a 4% mortality rate in monoarticular septic arthritis [1]. Diagnosis is usually based on clinical assessment followed by confirmatory synovial fluid analysis [1]. Septic arthritis typically presents with a single warm and painful joint and is accompanied by fever 30-40% of the time [2]. The treatment usually involves patients receiving two weeks of intravenous antibiotics, followed by four weeks of oral antibiotics, but can vary depending on the situation and the inciting organism. Septic arthritis most commonly occurs via a hematogenous spread of a microorganism that finds its way to the well-vascularized synovial capsule and penetrates it [3]. The most common pathogen causing septic arthritis is *Staphylococcus aureus* in both immunocompetent and immunocompromised patients [4]. Patients with asplenia have a reduced capacity to generate antibodies against the bacterial capsules; therefore, you see *Streptococcus pneumoniae*, *Salmonella*, *Haemophilus influenzae*​​​​​​, and particularly* Salmonella* spp. with sickle cell patients [5]. Historically, *H. influenzae* type b was the most common organism causing septic arthritis in children between one month and 24 months old [5]. Since the introduction of the type b *H. influenzae* vaccine, the cases of invasive typeable* H. influenzae* have dwindled. Rarely does *H. influenzae* affects the joint space in adults, but when it does, 95% of cases are reported to be of type b serotype [5]. For* H. influenzae *to infect a joint, the patient either did not receive a vaccination or, more likely, they are immunocompromised. Interestingly, there was a documented increase in cases of invasive *H. influenzae* infections in HIV patients in Atlanta, Georgia between 2008 and 2018, where 553 cases of invasive *H. influenzae *were found, but not specifically of septic arthritis [6]. We report a case where *H. influenzae* infects multiple joints in an individual diagnosed with HIV.

## Case presentation

**Figure 1:**
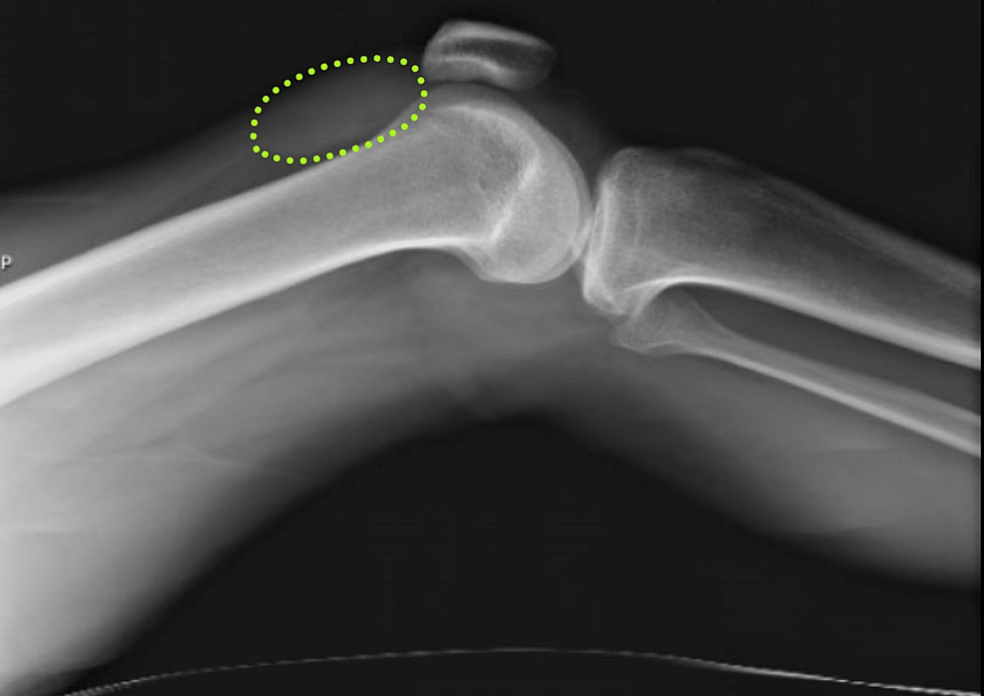
Initial knee X-ray revealing effusion (traced in green).

**Figure 2:**
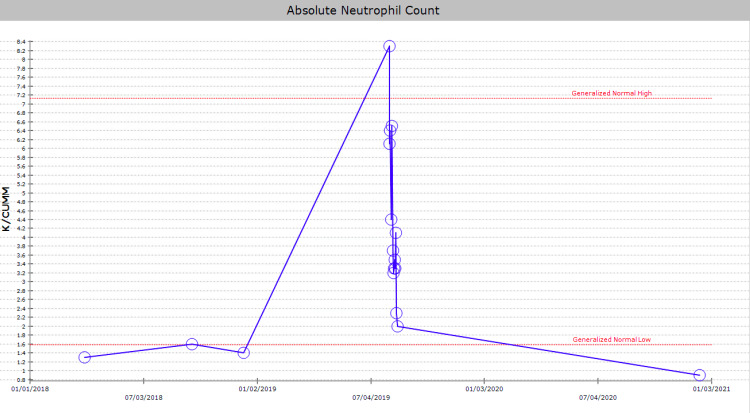
Absolute neutrophil count. This graph shows our patient with uncontrolled HIV with baseline neutropenia preceding this presentation, mounting a neutrophilic response to bacterial oligoarthritis. Blue line = absolute neutrophil count; K/CUMM = thousand cells per microliter; upper dashed red line = upper limit of normal; lower dashed red line = lower limit of normal.

**Figure 3:**
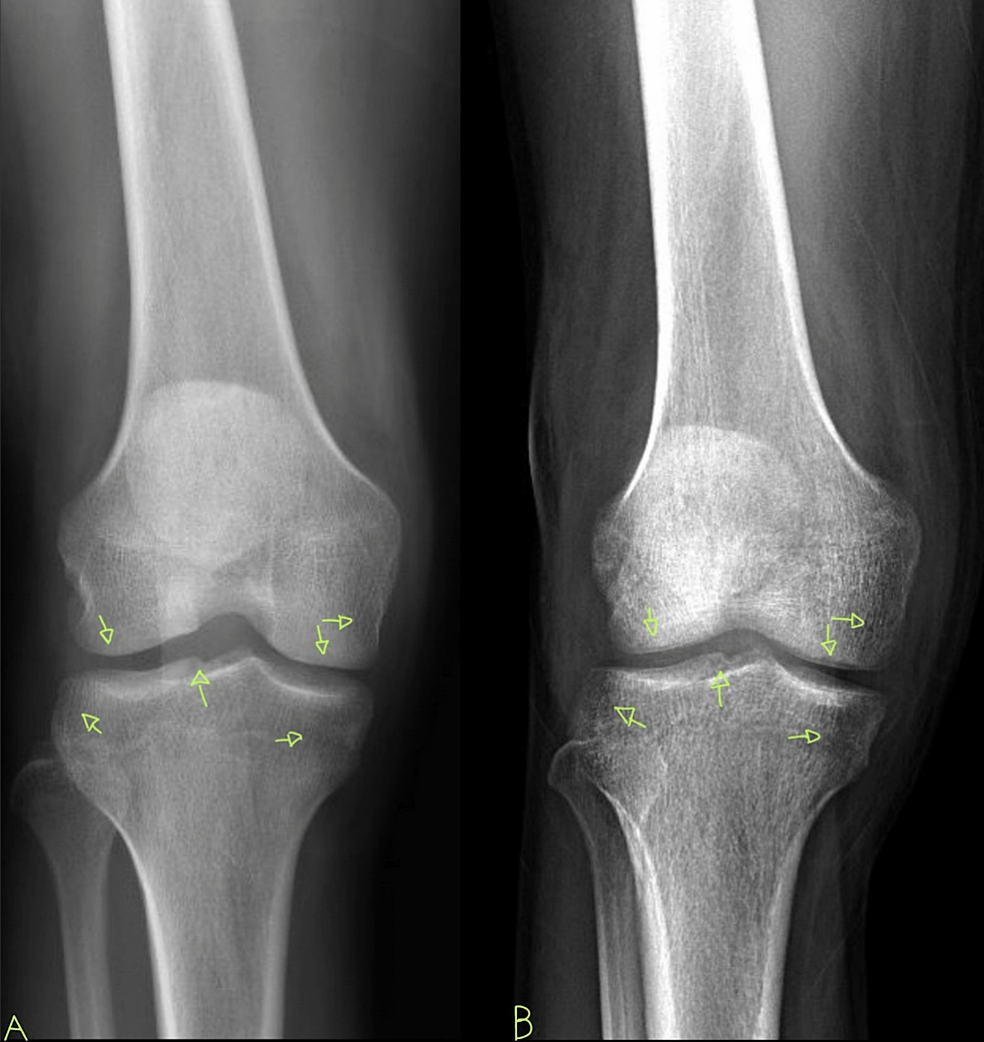
Pane A shows the initial knee X-ray. Pane B shows the follow-up knee X-ray with damage to the articular surface and evidence of osteopenia.

A 29-year-old African American male with a past medical history of HIV, not on treatment, presented to the emergency department with acute, asymmetric, oligoarthritis, complaining of the right knee and right shoulder pain that has progressively worsened over the past two weeks. He rated his pain as 10 out of 10, sharp, and non-radiating. He endorsed an episode of generalized malaise, subjective fever, chills, and weakness two weeks prior to admission that was self-resolved. His knee pain started after that, along with a decreased range of motion and difficulty ambulating. The patient also reported an episode of diarrhea one week prior to admission and reported being exposed to one of his colleagues with a flu-like illness. Diarrhea did not appear to be infectious but did raise concerns given his lack of HIV treatment as well as concerns for reactive arthritis given his associated joint pain. He was originally given steroids and discharged from the ED, only to return two days later with the progression of his previous pain. A review of systems was negative for oral and genital ulcers and skin lesions. On physical examination, his right knee was slightly edematous with mild erythema and tenderness to palpation. The right shoulder revealed a positive Hawkins and Neer test as well as reduced range of motion with tenderness to palpation. The patient also reported tenderness near the point of insertion of the right Achilles tendon. His last documented CD4 count was 321 cells/uL (cut-off for low CD4 is 580 cells/uL), and HIV1 RNA viral load was less than 20 copies/mL eight months prior to admission. His only documented medications included a combination of bictegravir, emtricitabine, and tenofovir pill daily that he was not adherent to and cyclobenzaprine as needed. He denied any drug allergies and was up to date with his immunizations. He was a waiter by occupation and he denied smoking or alcohol use, reported daily marijuana use, and had not recently traveled outside the United States. His initial vitals revealed he was afebrile, and his blood pressure and heart rate were within normal limits. His initial lab work showed an elevation in C-reactive protein (CRP) at 73 mg/L (cut-off for high CRP is 5.0 mg/L) and elevated erythrocyte sedimentation rate (ESR) at 99 mm/hr (cut-off for high ESR is 13.0 mm/hr). An X-ray of his right knee showed joint effusion (Figure 1). Initial presentation to the ED did reveal mild leukocytosis with neutrophilia (Figure 2) and monocytosis with a white blood cell (WBC) count mildly elevated at 11.2 k/cumm, neutrophilia of 8.3 k/cumm, and monocytosis of 1.5 k/cumm. After receiving glucocorticoids and returning two days later, the patient's leukocytosis quickly transitioned to leukopenia, WBC count was 9.7 k/cumm, and neutrophil count was 6.4 k/cumm but showed monocytosis with an elevated count of 1.7 k/cumm. Initial CD4 count was low and lymphocyte count was on the lower limit of normal that corresponded with uncontrolled HIV infection in the setting of non-adherence to antiretroviral therapy (ART). Arthrocentesis was performed on the right knee producing an unspecified amount of turbid yellow synovial fluid and analysis for cell count revealed a nucleated cell count of 7,556/cumm and RBC count of 1,555/cumm. Right knee synovial fluid culture revealed numerous polymorphonuclear neutrophils (PMNs) and grew a moderate amount of *H. influenzae*. He was admitted to the hospital for septic arthritis and started on intravenous ceftriaxone. Orthopedic surgery was consulted and performed an incision and drainage of the right knee but was unsuccessful in obtaining fluid from the right shoulder. Rheumatology was consulted given the patient's report of multiple joints being involved. Rheumatology recommended workup for spondyloarthritis, human leukocyte antigen B27 (HLA-B27), reactive arthritis, rheumatoid factor (RF), and HIV-related arthropathy workup, given the patient's low CD4 count. RF was slightly elevated at 17 IU/mL (cut-off for high RF is 15 IU/mL), and HLA-B27 gene testing was negative. Infectious disease (ID) was consulted for bacteremia and septic arthritis. ID recommended starting ceftriaxone, discontinuing vancomycin, and discharging the patient with six weeks of azithromycin 500 mg daily once blood cultures were negative. No susceptibilities were obtained. No glucocorticoids were given during his hospitalization, as he was immunocompromised and did not respond to glucocorticoids after his initial presentation to the emergency department. The patient was discharged with instructions to complete his antibiotic regimen and return to the hospital if his symptoms worsened. His cultures did not grow other pathogenic organisms, and he returned for a one-month knee X-ray that revealed articular damage and osteopenia (Figure 3A). The patient was lost to follow-up with the infectious disease, orthopedic surgery, and rheumatology outpatient following his last knee X-ray one month after initial presentation (Figure 3B).

## Discussion

Within the human body exists several hermetically sealed “sterile zones,” such as the inside of the brain, the eye, and the joint spaces. Healthcare providers should be alarmed when a typically noninvasive and docile bacteria, such as *H. influenzae*, not only invades the bloodstream but also happens to violate a sterile zone such as the joint space, as seen in our patient. A proper immune system would easily ward off the invasion of the joint space, particularly by such docile bacteria as *H. influenzae, *which has limited virulence capabilities.

The joint space is a sterile space that can be classified into two broad categories: solid joints (nonsynovial) and cavitated joints (synovial). Nonsynovial joints, referred to as synarthroses, have minimal extra space within them, are immobile, and are designed to provide the human organism with structural integrity. Nonsynovial joints are rarely, if ever, implicated in documented cases of septic arthritis and do not concern us for our topic. However, synovial joints have extra space inside them and are designed to provide the human body with mobility. It is the extra space within synovial joints that is of particular interest to us, as it provides a breeding ground for infiltrating bacteria, much like a culture medium would. The synovial membrane wraps around the joint to create a sealed-off and sterile environment and is lined by type A synoviocytes and type B synoviocytes. Type A synoviocytes are like macrophages in activity, and type B synoviocytes synthesize hyaluronic acid and other proteins and are similar to fibroblasts in their activity. Bacteria can facilitate one of five routes to reach joint synovium, the most common being hematogenous spread [7]. Other routes bacteria can use include joint capsule invasion from adjacent muscles, ligaments, or bones, or via trauma or diagnostic procedures [7]. Risk factors that increase the likelihood of septic arthritis include aging, antibiotic resistance, orthopedic procedures, immunosuppressive drugs or HIV infection, diabetes, leukemia, cancer, hypogammaglobulinemia, cirrhosis, granulomatous disease, and IV drug use [7].

Neutrophils are an integral component of the body’s immune system as they are first-line responders against invading foreign bacteria, fungi, and protozoa and have various tools in their arsenal to combat pathogens such as *H. influenzae*. When a sterile zone is violated, a metronomic cascade of interleukins and paracrine signaling molecules initiates a systematic chain of events that results in an inflammatory response against the offending pathogen. Neutrophils are myeloid leukocytes that are part of the innate immune system and comprise 50-70% of all circulating leukocytes in humans [7,8]. They are so important that 55-60% of the bone marrow is specifically dedicated to generating neutrophils daily [8]. As a critical component of the immune system, even a slight decrease in the systemic circulation of neutrophils can render hosts immunocompromised [8].

Some of the mechanisms utilized by neutrophils can act as a double-edged sword with respect to surrounding tissues. For example, the neutrophil extracellular traps (NET), whereby neutrophils eject their DNA and histones and create a barbed wire-like web around offending pathogens as both parties die [7]. Oxidative bursts are enzymatic reactions whereby neutrophils utilize the functions of atomic physics (electrons) to kill invading pathogens. Reactive oxidative species (ROS) are notorious for wreaking havoc on all living tissues, even if they are produced by the immune system on purpose. Collateral damage includes neutrophils damaging the very joint they are trying to save and eroding the structural integrity of the joint with electron bursts designed to blow fatal holes in the bacterial cell wall [7].

Once HIV renders the immune system dysfunctional, it is well documented that opportunistic offending pathogens wreak havoc in areas where they normally would never be found. *H. influenzae* is a bacterium that is usually found in the nasopharynx and upper airway tract, and thanks to vaccination, it rarely causes clinically notable infections. *H. influenzae* is not high on the differential list of organisms when considering any systemic infection, let alone polyarticular bacterial septic arthritis. Once *H. influenzae* infiltrates the synovium, the synovial fluid's low shear environment is unlike fast-moving blood and this allows bacteria such as *H. influenzae* to facilitate microbial surface components recognizing adhesive matrix molecules to gain a foothold and begin to multiply itself inside the joint space [7]. The synovium in a healthy subject responds by inducing hyperplasia of its inner layer to recruit macrophages and secretes interleukin-8 (IL-8) to recruit neutrophils as its first line of defense [7]. Neutrophils are indispensable first-line responders, and we can see how compromise in their function can increase the likelihood of bacteria setting up "camp" inside the joint pace unabated.

Patients afflicted with HIV have multifactorial neutropenia secondary to viral toxicity of hematopoietic stem cells, myelotoxic ART, as well as increased strain on the overall immune system due to infections and malignancies [9,10]. HIV-induced toxicity to T-cells reduces granulocyte-macrophage colony-stimulating factor (GM-CSF), which is the primary and necessary stimulant to start the production of neutrophils in the bone marrow [9,10]. In addition to decreased GM-CSF, HIV has been shown to directly attack hematopoietic stem cells via direct infection and Fas-dependent apoptosis. HIV proteins suppress the proliferation of granulomonocytic progenitor cells and induce Fas-dependent apoptosis of hematopoietic stem cells that would otherwise mature into neutrophils [9,10]. Also, polyclonal B-cell activation has been shown to produce anti-neutrophil antibodies that further exacerbate neutropenia [11,12]. By upregulating programmed death-ligand 1 (PD-L1) expression on peripheral neutrophils, HIV causes PD-L1-dependent suppression of CD4+ T-cells, which leads to both T-cell exhaustion as well as increased suppression of CD4-mediated neutrophil dysfunction [9,13]. Therefore, the collapse of adaptive immunity in conjunction with compromised innate immunity allows for otherwise harmless pathogens to mount a fatal infectious attack on the host. Patients with HIV infection have decreased peripheral neutrophil count and reduced antimicrobial neutrophil function, predisposing patients to increased risk of secondary infections.

Aside from the frank quantitative neutropenia induced by HIV, the actual qualitative function of the neutrophils also becomes compromised. With oxidative bursts, chemotaxis, and compromised NET activity in patients with HIV, ART seems to not help but worsen this phenomenon [9]. ART does not improve neutrophil function but rather creates impotent neutrophils that are ultimately useless vis-à-vis polyarticular bacterial septic arthritis. The combination of HIV and ART hinders the baseline function of neutrophils and then results in a large surge of hyperactive, trigger-happy, and impotent neutrophils. When the deranged neutrophils of HIV-infected patients on ART arrive to the joint space, they will likely release an unnecessary amount of ROS across the joint space as they have lost their ability to calibrate their oxidative bursts [9]. It has been proven that neutrophils in the peripheral blood of ART-treated, HIV-infected individuals express reduced levels of L-selectin (CD62L), FcgRIIb (CD16), and increased integrin (CD11b) [9]. This expression pattern renders neutrophils “hyperactive” and is accentuated by inflammatory comorbidities [9]. This results in not clearing the infection properly and allowing for opportunistic infections to continue to flourish unabated as well as excessively damaging the joint space. This damage can be appreciated in our case report in Figure 3B.

Neutropenia is thought to accompany HIV infection in approximately 10-50% of patients and was noted in our patient's initial presentation (Figure 2) [14]. The cutoff for true neutropenia in HIV-infected individuals has been debated but is generally accepted at approximately 2,000 cells/microliter (versus 3,500 cells/microliter in immunocompetent hosts). A recent, large cohort study of HIV-infected women discovered a baseline of 44% of HIV-infected women had neutrophil counts less than 2,000 while a 7.5-year follow-up period showed 79% of HIV-infected women had neutrophil counts below 2,000 cell/microliter [14]. Therefore, physicians might even consider different cut-offs for neutropenia in patients with uncontrolled HIV infections.

## Conclusions

This patient’s rare presentation served as a great learning opportunity and serves as a reminder that HIV does not only affect lymphocyte function. This case highlights the collateral damage to the joint space that comes part-and-parcel with septic arthritis and why it should be taken seriously. We see the comprehensive suppressive effects of HIV on myeloid precursors within the bone marrow to change the expression of signaling molecules on neutrophils. Not only does HIV attack neutrophil function at its core, but should a neutrophil make it through this barrage of assaults, it will either be impotent or hyperactive, resulting in a lose-lose situation. Interestingly, ART further compromises neutrophil function. We see how the effects of glucocorticoids and the resumption of ART could further complicate the treatment course. The patient was not adherent to ART prior to admission and was initiated during the second day of hospitalization. This case is also a reminder of the judicious use of glucocorticoid therapy when patients with HIV present with joint pain. The neutropenic effects of HIV infection and ART can be particularly concerning when it comes to treating acute infections and should be taken into consideration when patients present with low CD4 counts and are non-adherent to ART.
